# Quantitative evaluation of science and technology financial policies based on the PMC-AE index model: A case study of China’s science and technology financial policies since the 13th five-year plan

**DOI:** 10.1371/journal.pone.0307529

**Published:** 2024-08-01

**Authors:** Hongyuan Shen, Panyu Xiong, Linfeng Yang, Ling Zhou

**Affiliations:** 1 School of Economics and Management, Southwest University of Science and Technology, Mianyang, China; 2 China Three Gorges Group Basin Management Center, Yibin, China; University of Kragujevac: Univerzitet u Kragujevcu, SERBIA

## Abstract

The formulation of science and technology financial policies directly influences the direction of national economic development. Quantitative evaluation of these policies is an important method to reflect the consistency and strengths and weaknesses of policy interrelations. This paper analyzes 16 science and technology financial policy documents issued by the Chinese central government from 2016 to 2022, using text analysis and content analysis to extract keyword frequencies, and constructs 9 primary variables and 34 secondary variables. For the first time, a PMC-AE index model for science and technology financial policies is established, and a quantitative evaluation is conducted on 5 significant policy documents out of the 16. The results show that, from an overall analysis, Policy 1 and Policy 4 are at a good level, while the other three policies are at an excellent level. From the analysis of individual policy PMC-AE indexes, the rankings in descending order are: P2 > P5 > P3 > P4 > P1. Overall, the policies effectively meet the needs of China’s science and technology financial development, with P2, P3, and P5 being at an excellent level, P4 at a good level, and P1 at an acceptable level, mainly reflecting the need for improvement in aspects such as policy synchronization with the current stage, targeted entities, guiding fields, and policy content. It is recommended that Chinese government departments should focus on five aspects in policy formulation: building a talent system for science and technology finance, improving the quality of financial services, coordinating central and local financial policies, protecting intellectual property rights in science and technology finance, and strengthening financial supervision. This will be conducive to the effective implementation of science and technology financial policies.

## Introduction

As society progresses, the economy develops, and technological advancements have given rise to a series of financial instruments serving various aspects of our lives, making significant contributions to the nation’s economic take-off and people’s well-being. The organic integration of the Internet+ and artificial intelligence has accelerated the application of science and technology financial services in various social fields. At the same time, it has also brought certain risks and challenges. How to assess the security and guidance of current science and technology financial policies is a difficult question that lies before us. Looking back at the implementation history of China’s science and technology finance, it can be divided into three periods: the nascent period (1985–2008), the initial stage (2009–2014), and the rapid development period (2015–2024). During the nascent period, the overall characteristic of China’s financial technology was the "technology-first" + "government-led" model, where the government was the main driver of financial support for technology, primarily through increased fiscal spending in the field of science and technology and proposing a series of science and technology financial measures such as guiding financial institutions to increase investment in high-tech industries and enhancing fiscal support for technological innovation. In the initial stage, the overall characteristic was the "financial empowerment" plus "regional pilot" model, which for the first time clearly defined science and technology finance, pointing out that innovations in various financial tools such as credit, investment, and insurance promote technological development, committed to broadening the funding sources for technology enterprises, constructing a multi-level capital market, and formulating a series of science and technology financial policies conducive to "technological empowerment". During the rapid development period, the overall characteristic of science and technology finance was "integration of science and finance" plus "multi-stakeholder participation, multi-level market, diversified tools". In this period, the focus of science and technology financial policies shifted from constructing to perfecting the science and technology financial system, with corresponding policies being more regional, targeted, and innovative. Looking at the three important periods of China’s science and technology finance, the evolution of policies has shown a transformation from abstract to concrete, from discrete to comprehensive, and from fragmented to integrated. In this process, the Chinese government or departments have issued a series of guiding policies on science and technology finance, clarifying specific contents, such as in 1993 when the State Council issued the "Decision of the State Council on Financial System Reform". In 2014 and 2016, the content of financial technology was clarified in the form of government reports and the "13th Five-Year" Science and Technology Innovation Plan, and in 2019, the first top-level document "Financial Technology Industry Development Plan (2019–2021)" was released. Following this, six national ministries approved a one-year financial technology application pilot in 10 places including Beijing, Shanghai, and Sichuan. The pilot cities subsequently introduced financial technology policies with the aim of promoting the development of science and technology finance to improve quality and efficiency. According to statistics from the China Banking and Insurance Regulatory Commission, as of July 28, 2023, China has established over 1000 science and technology branches and specialized science and technology financial institutions. According to data released by the People’s Bank of China, as of the end of June 2023, the balance of medium and long-term loans in the high-tech manufacturing industry reached 2.5 trillion yuan, with a year-on-year increase of 41.5%, maintaining a high growth rate of over 30% for three consecutive years, and the role and influence of science and technology finance have gradually become apparent.

Up to now, the discussions on science and technology finance among policy makers, academia, and industry have mostly focused on how science and technology finance supports technology enterprises and innovation. However, there is still a lack of research findings on the evaluation of the effectiveness of science and technology financial policies. In light of this, it is very urgent to study the effects of financial technology-related policies, which plays a crucial role in guiding China’s science and technology financial market. Currently, science and technology financial policies cover a wide range of content: this includes policy effectiveness, whether policy supervision is in place, the consistency of related policies, the level of policy issuance, knowledge, property rights, financial talent, financial intermediaries, and more. Based on the collation of science and technology financial policy documents, this paper explores whether the execution effects of science and technology financial policies are reasonable and can have policy guidance. It conducts scientific quantitative analysis and evaluation research, which is of great practical significance for the steady advancement of China’s current science and technology financial policies, solving the problems of unbalanced and insufficient development of financial technology, and improving the policy and regulatory standards system.

The main sections of the paper are arranged as follows: the first part is literature review, the second part is materials and methods, the third part is quantitative evaluation of science and technology financial innovation policies, the fourth part is discussion, and the fifth part is research conclusions and recommendations.

## Literature review

Looking back at the research achievements in science and technology finance, Schumpeter [1] was one of the first to propose that the new economy requires the reintegration of a series of newly formed production economic elements and external conditions into the existing production and operation system to stimulate economic development, which then leads to financial innovation. With the development of the social economy [2], technological innovation has become the new engine of social development. Combining it with financial innovation can further promote the development of emerging industries. However, market failures may occur during the development of emerging industries. At this time, timely government intervention in the market economy, macroeconomic regulation, and the introduction of corresponding industry regulatory policies to improve "general policies + specific policies" measures in response to market failures can not only solve the problems of limited growth of a large number of small and medium-sized enterprises in the post-pandemic era and optimize the national innovation environment but also achieve market supply and demand balance and improve the efficiency of resource allocation [3]. Reviewing the science and technology financial policies introduced since the reform and opening up, it has been found that these policies have been analyzed from different perspectives [4], and research methods such as co-word analysis and social network mapping have been used to study the policy theme changes in science and technology finance [5]. Research findings also indicate that there are differences in the implementation effects of science and technology financial policies on enterprises of heterogeneous nature and market types [6].

With the rapid development of China’s economy, various regions across the country have successively introduced related science and technology financial policies. A considerable amount of research has been conducted by scholars on how to measure the implementation effects of these policies. For instance, the level of development of science and technology finance in Guangdong Province has consistently been at the forefront nationally, but the problem of uneven development within the province is severe [7]. The provincial capital, Guangzhou, places great emphasis on the integration of science and technology with finance, achieving leapfrog development, thereby leading the way in the level of science and technology financial development. In contrast, some other areas have less than ideal development in financial technology [8]. In some provinces, due to a lack of emphasis on the integration of science and technology with finance, not only are there disparities in economic development levels between provinces, but significant differences also exist within the regions of each province. Local governments should analyze specific problems in detail and formulate a series of policies suitable for the local development of science and technology finance. Taking Jiangsu Province as an example, the provincial government has played different roles in financing roadshows for science and technology finance projects, thus alleviating the financing difficulties of technology-based SMEs and creating broad development space for them [9]. Besides, although some regions have a relatively complete system of science and technology financial policies, the synergy between these policies is not strong, such as in Shanghai [10]. Some areas even experience a general situation where coordinated development and policy impact efficiency are both low, such as in Hebei Province [11]. So, how to solve the problem of policy impact efficiency? Pang and Zhang [12] believe that by strengthening the coordination horizontally and vertically among departments, the efficiency of policy transmission from the provincial level to the municipal level can be improved. Given the differences in development among China’s provinces, those with lower development efficiency can formulate local policies based on regional development advantages and find paths that are conducive to their own efficient development of science and technology finance [13].

For a long time, the geographical position of the eastern region has been superior to that of the western region, and the eastern coastal areas, with their numerous ports, facilitate trade with countries around the world. Not only do the eastern coastal cities have advantageous geographical locations, but they also place emphasis on the development of financial technology, becoming economically developed regions [14]; whereas in the western region, cities such as Chengdu and Chongqing, despite having introduced corresponding policies for technological innovation to support technology enterprises and build innovation platforms, still exhibit low efficiency in science and technology finance [15]. In contrast, some western provinces with geographical environments slightly inferior to Chengdu and Chongqing, such as Jiangxi and Guizhou, have low levels of development in science and technology finance, yet the growth rate of their financial efficiency is fast. Huang and Qiu [16] evaluated the efficiency of China’s science and technology finance, suggesting that science and technology finance overall does not significantly enhance technological innovation and efficiency. With the strengthening of centrality in the capital network, macro-orientation, financial support, and policies for technical and financial patent protection will significantly improve the innovation performance of enterprises [17]. Science and technology financial policies can be used to promote enterprises in pilot cities, enhance innovation efforts, and improve innovation performance, accelerating the transformation and upgrading of industrial structures [18–20]. In line with the development requirements of China’s "13th Five-Year Plan," the financial industry should first meet the development needs of real enterprises and accelerate the construction of new science and technology financial service platforms [21]. As China’s economic development enters a new normal, practicing green finance and the concept of low-carbon development, science and technology finance has accelerated the process of high-quality economic development in China [22–25], improving the investment efficiency of corporate green innovation [26] and the driving effect of green development [27]. Meanwhile, the promoting effect of green technology innovation in science and technology finance exhibits spatial heterogeneity, with the eastern region showing an increasing marginal effect, and the central region displaying an inverted "U" characteristic [28], which is conducive to the risk resistance level of enterprises [29], providing strong support for the development of new productive forces [30]. However, some scholars believe that the excessive financialization of science and technology finance may have a negative impact on the real economy [31].

Policy evaluation not only has a direct promoting effect on policy formulation, implementation, and feedback adjustment [32], but also has a significant impact on the understanding of global trends and the establishment and adjustment of national science and technology financial strategies [33]. Policy evaluation originated with Suchman’s [34] five categories of evaluation, followed by Poland’s [35] "Three E’s" evaluation framework and Wollmann’s [36] classical policy evaluation. With the continuous exploration of policy evaluation methods, many methods of policy assessment have emerged. Shen et al. [37] used the difference-in-differences approach to empirically analyze the policy effects of the energy rights trading system, and applied the Analytic Hierarchy Process (AHP) to evaluate China’s military-civil fusion policies [38]. Feng et al. [39] constructed a BP neural network-based model for predicting carbon emissions in the electricity sector; some scholars used the Delphi method and Fuzzy Analytic Hierarchy Process (FAHP) to assess the main factors and sub-factors of green finance [40]; analyzed the risks related to China’s green infrastructure financing and prioritized them [41], as well as evaluating the environmental, social, and governance (ESG) factors and policy choices in China’s green finance investment decisions [42]. Currently, many evaluation methods have certain flaws, mainly reflected in the inevitable subjectivity and lower precision. The PMC index model method, however, obtains original data through text mining, which can largely avoid subjectivity and improve the precision of policy evaluation. The PMC method is based on the Omnia Mobilis (everything is in motion) assumption proposed by Ruiz Estrada et al. [43] when studying the application in Cartesian space, and has constructed the PMC index model, innovating the single policy evaluation method. This method judges the consistency of policies from a multidimensional perspective and directly observes the strengths and weaknesses of policy texts by constructing the concave and convex shapes of the PMC surface [44, 45]. Since then, this method has been widely applied to policy evaluation research: for example, in the construction industry [46]; nursing insurance policies [47] and farmland protection policies [48]; China’s pork industry [49]; the efficiency of green development in the Yangtze River Economic Belt [50]; China’s watershed ecological compensation policies [51] and the effectiveness evaluation of provincial public funding policies for private colleges in China [52]. The PMC method has been used to study the effectiveness of related policies, providing a good evaluation of the consistency and deficiencies of different policies during the specific implementation process.

In summary, scholars have made rich comparisons of the results from the perspective of the role of science and technology financial policies in promoting economic development, as well as the changes in the policy themes of science and technology finance, with a focus on elucidating the impact of science and technology finance on regional economic development and revealing the problems of unevenness and low efficiency in policy execution across different regions. With the robust development of China’s economy, the Chinese government or relevant departments have introduced a series of policies emphasizing the importance of science and technology finance, making the evaluation of the effectiveness of these policies particularly urgent. There are many existing evaluation methods, each with its own shortcomings. The Analytic Hierarchy Process (AHP) analyzes the correlation of a limited number of variables in a time series, which is limited by the number of variables and neglects the influence of some factors that are small yet actually present. The subjectivity of the Fuzzy Comprehensive Evaluation and Grey Comprehensive Evaluation methods is strong, and their resolution is poor [53].

Although the PMC method has been widely used in evaluating policies in many fields, its application in the evaluation of China’s science and technology financial policies is still a blank area. This paper attempts to use the PMC method to evaluate the effectiveness and consistency of science and technology financial policies, thereby analyzing whether the policy itself has any shortcomings in its guiding effectiveness. In view of this, the marginal contribution of this paper lies in: first, constructing for the first time a PMC-AE index model to quantitatively evaluate the science and technology financial policies issued by the central government since the "13th Five-Year Plan" of China, by extracting high-frequency words to construct 9 primary variables and 34 secondary variables, and establishing a multiple input-output table; second, judging the policy execution effects based on the intuitive concave and convex shapes of the PMC surface, and evaluating the consistency and effectiveness of science and technology financial policies; third, proposing five reasonable suggestions for the central government functional departments formulating science and technology financial policies. This is conducive to the Chinese government’s revision of science and technology financial policies, improving the policies, and enhancing the execution effects of science and technology financial policies.

## Materials and methods

### Mechanism of action for science and technology financial policy

**Fig 1:**
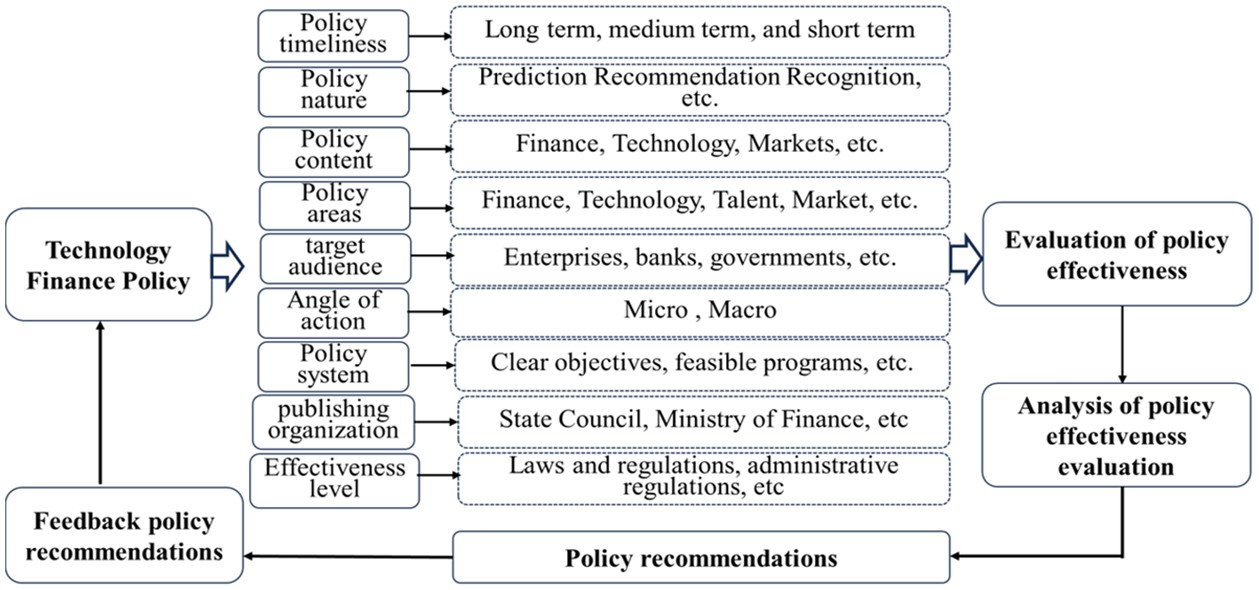


Once the science and technology financial policy is enacted and implemented, it will have significant impacts on the economy, society, technology, politics, and the environment. During the implementation of the science and technology financial policy, it is influenced by factors such as the market, regulation, laws and regulations, timeliness, and talent. This also reflects a series of issues. By analyzing and judging the effectiveness of policy implementation, constructive feedback can be provided to the relevant departments. This is conducive to the further improvement of science and technology financial policies and promotes national economic development, thereby forming a complete cycle chain, as shown in Fig 1.

### Statistical analysis

#### PMC-AE index model

**Fig 2:**
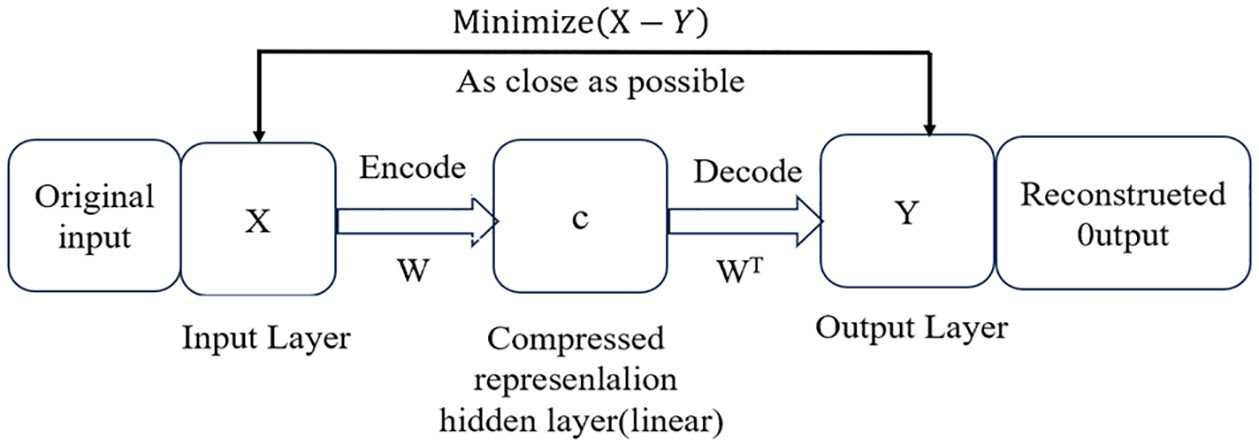


The difference between the PMC-AE index model and the PMC index model lies in the method of index calculation. The former uses acoustic emission technology for parameter fusion, which has advantages over the latter’s linear fusion. An Auto-Encoder is an unsupervised learning model. Based on the backpropagation algorithm and optimization methods (such as gradient descent), it uses the input data X itself as supervision to guide the neural network to try to learn a mapping relationship, thereby obtaining a reconstructed output Y. The principle is that it first compresses the input into a latent space representation, forming a hidden layer C (with spatial representation characteristics), and then reconstructs this compressed spatial representation into output, similar to the principle of the commonly seen Principal Component Analysis (PCA) method. During the encoding process, the Auto-Encoder can represent both linear and nonlinear transformations, while PCA can only perform linear transformations. The vector of its hidden layer has the effect of dimensionality reduction. Essentially, an autoencoder is a data compression algorithm, with both compression and decompression algorithms implemented through neural networks. Building an autoencoder requires two parts: an encoder and a decoder. The encoder compresses the input into a latent space representation, creating a hidden layer (or multiple hidden layers) that contains a low-dimensional vector of the input data’s meaning. It can be represented by the function f(x). The decoder reconstructs the latent space representation back into output, that is, reconstructs the input data through the low-dimensional vector of the hidden layer. It can be represented by the function g(x), and both the encoding function f(x) and the decoding function g(x) are neural network models. As shown in Fig 2, the encoding process of the original data X from the input layer X to the hidden layer C is:

C=Φf(x)=σ(Wx+b) (1)

The decoding process from the hidden layer C to the output layer Y is:

$$Y=\Phi g(c)=\sigma (W^{T}c+B)$$ (2)

Then, the optimization objective function of the algorithm is written as:

$$Minimize\;Loss=dist\left(X,\;Y\right)$$ (3)

Where *dist* is the distance measurement function between the two, usually represented by MSE (Mean Squared Error).

In the aforementioned formula, *X* = (*x*_1_, *x*_2_, *x*_3_ ⋯, *x*_*n*_)^*T*^ represents the established multi-dimensional indicators for evaluating science and technology financial policies, while *Y* = (*y*_1_, *y*_2_, *y*_3_ ⋯, *y*_*n*_)^*T*^ denotes the corresponding values of the output layer nodes. The activation functions for the hidden layer and output layer, denoted by f and g respectively, are typically Sigmoid, Tanh, or Softplus functions, among others, and may be identical or distinct. *C* = (*c*_1_, *c*_2_, *c*_3_ ⋯, *c*_*m*_)^*T*^ signifies the values of the hidden layer nodes; W and W^T^ represent the weight matrices between the input and hidden layers and between the hidden and output layers, respectively. The dimensions of these matrices are determined by the number of neurons in the preceding layer (rows) and the subsequent layer (columns). The bias vectors *b* = (*b*_1_, *b*_2_, *b*_3_ ⋯, *b*_*m*_)^*T*^ and *B* = (*B*_1_, *B*_2_, *B*_3_ ⋯, *B*_*n*_)^*T*^ pertain to the input-to-hidden and hidden-to-output layer transitions, with dimensions corresponding to the number of nodes in the subsequent layer neurons. The objective of Autoencoder (AE) training is to minimize the discrepancy between Y and X [54].

When the dimensionality of the original data is excessively high, or when a lower-dimensional representation is required, the neural network architecture can be modified by increasing the number of layers and progressively reducing the number of neural unit nodes. Consequently, after training, X is transformed into C through a nonlinear combination, C is then transformed into Y through another nonlinear combination, with the aim of achieving Y = X. Hence, C can be regarded as the nonlinear embodiment of X, and Y is derived from the decoding of C. Thus, C can serve as the composite score of policy texts after the integration of various indicators [55].

**Fig 3:**
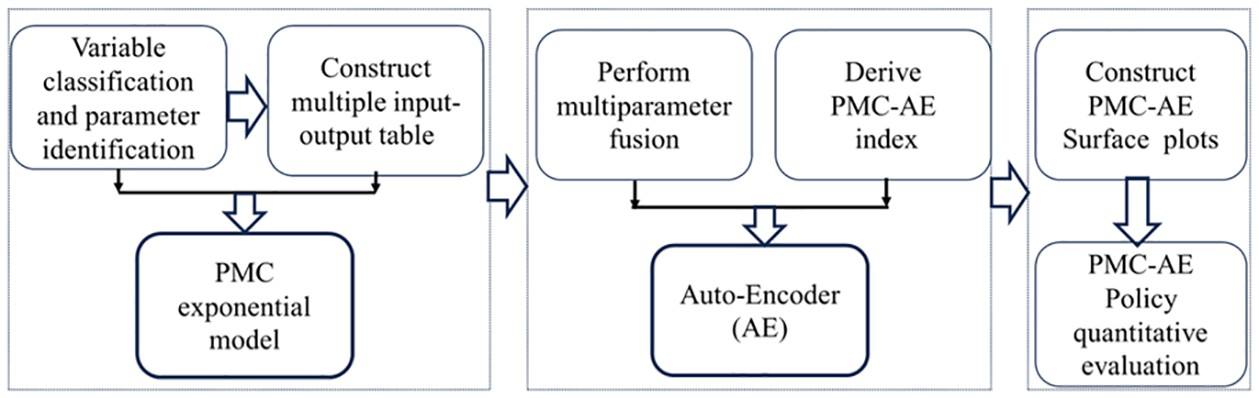


The construction of the PMC-AE index model mainly consists of the following four basic steps: (1) Classification of variables, calculation, and determination of model parameters. (2) Establishment of a multi-parameter input-output table for science and technology financial innovation policies, and assignment using text refinement techniques. (3) Fusion of multiple parameters using the autoencoder technology of neural networks to calculate the PMC-AE index. (4) Drawing of the PMC-AE index surface to evaluate the merits of science and technology financial innovation policies. The principle of constructing the PMC-AE index model is shown in Fig 3.

#### Data acquisition and preparation

This article takes the beginning year of China’s "Thirteenth Five-Year Plan" as the base point, mainly selecting relevant policies on science and technology financial innovation issued by the Central Committee of the Communist Party of China, the State Council, and the innovation demonstration zones. The policy data sources include official websites such as the State Council, the People’s Bank of China, Shanghai, Guangzhou, and the Shenzhen Special Economic Zone. By collecting, organizing, and screening, we ensure the accuracy of the policy texts.

To ensure that the content of policy information is consistent with the theme of science and technology financial innovation, the following principles were adhered to when organizing and selecting policy texts: First, choose policies closely related to science and technology financial innovation, excluding those that only mention science and technology financial innovation. Secondly, the types of texts are general document management systems that have been published, involving outlines, plans, proposals, opinions, suggestions, notices, etc. To ensure the timeliness of policy research, 16 policy documents related to science and technology financial innovation were ultimately selected to construct a database of policies on science and technology financial innovation (see Table 1).

**Table 1:** Science and technology financial policies issued by the central government from 2016 to 2022.

ID	Policies	Year
P1	Notice on Promoting and Supporting Innovative Reform Measures	2017
P2	Notice on Issuing the Plan for Further Deepening Reforms and Opening Up of the China (Guangdong) Free Trade Pilot Zone	2018
P3	Notice on Promoting the Second Batch of Supportive Measures Related to Innovation Reforms	2019
P4	Several Opinions on Strengthening Financial Services for Private Enterprises	2019
P5	Outline Development Plan for the Guangdong-Hong Kong-Macao Greater Bay Area	2019
P6	Guiding Opinions on Promoting the Healthy Development of Small and Medium-sized Enterprises	2019
P7	Opinions on Establishing a More Perfect System and Mechanism for Market-based Allocation of Factors	2020
P8	Implementation Plan for Comprehensive Reform Pilot of Building Shenzhen into a Demonstration Area of Socialism with Chinese Characteristics (2020–2025)	2020
P9	Suggestions on Formulating the Fourteenth Five-Year Plan for National Economic and Social Development and the Long-Range Objectives Through the Year 2035	2021
P10	Action Plan for Building a High-Standard Market System	2021
P11	Opinions on Supporting Pudong New Area to Achieve High-Level Reform and Opening-Up and to Build a Leading Area for Socialist Modernization	2021
P12	Overall Plan for Deepening the Reform and Opening Up of the Qianhai Shenzhen-Hong Kong Modern Service Industry Cooperation Zone	2021
P13	Opinions on Promoting High-Quality Development of the Central Region in the New Era	2021
P14	Opinions on Comprehensive and Accurate Implementation of the New Development Concept to Achieve the Peak of Carbon Dioxide Emissions and Carbon Neutrality	2021
P15	Financial Technology Development Plan (2022–2025)	2022
P16	Opinions on Accelerating the Construction of a Unified National Market	2022

#### Policy content keyword

**Fig 4:**
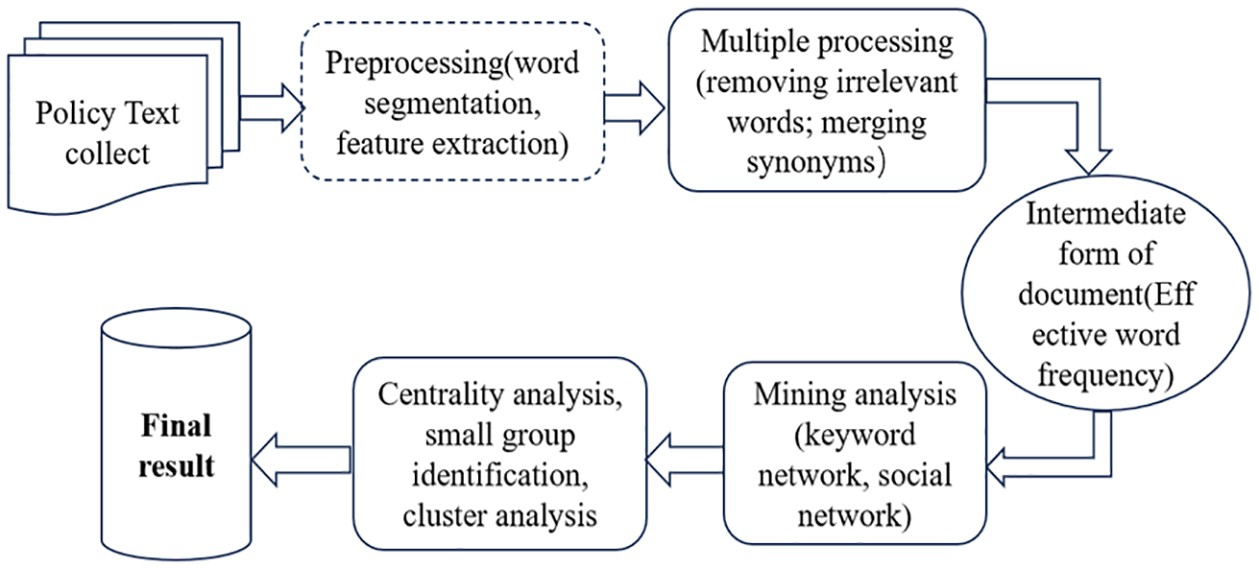


**Fig 5:**
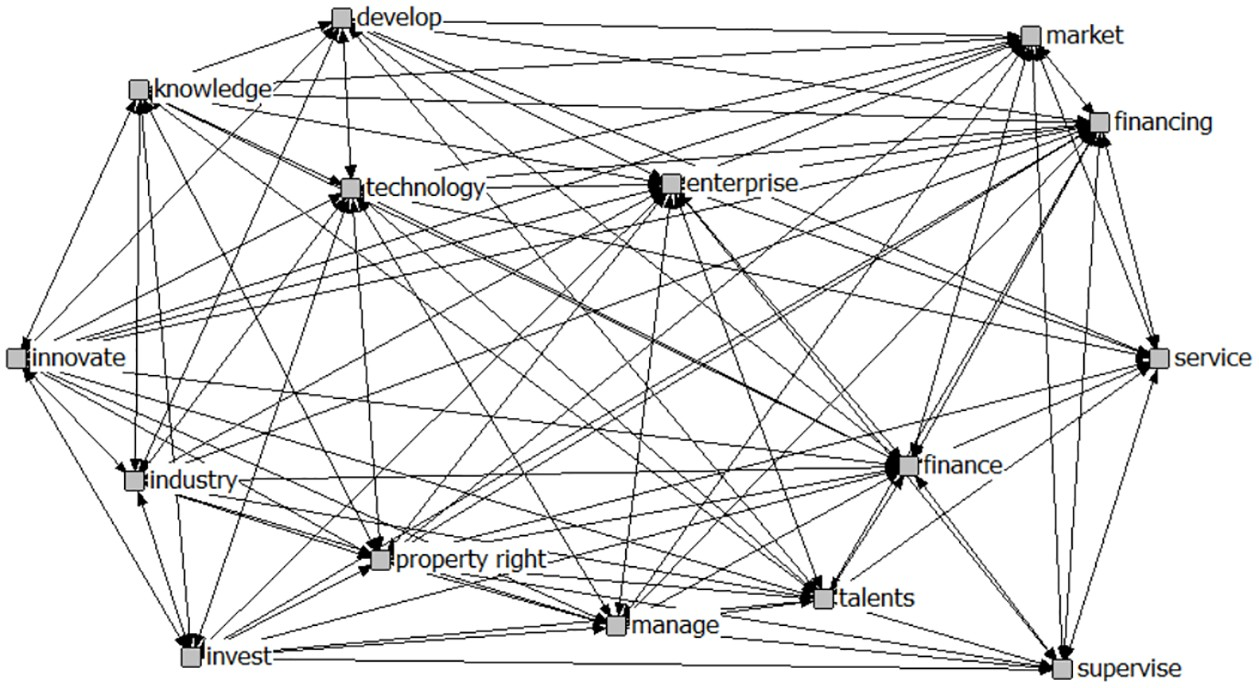


In recent years, many citation analysis software tools such as CiteSpace and VOSviewer have emerged domestically and internationally. These tools can construct a comprehensive knowledge unit of literature, informing us of the key research areas in a discipline. However, such citation software has a problem of being "too broad" in determining research content. ROSTCM, on the other hand, can analyze the key research content of a field in more detail by capturing literature abstracts. The software is powerful and easy to operate, and the text mining process is shown in Fig 4. Based on the research results of previous scholars [56], and in combination with the mechanism of action of science and technology financial policies mentioned above, this paper has established 9 primary indicators that are broad and universal. The setting of secondary indicators is through comprehensive reference to relevant domestic and international literature [57], and in conjunction with the specific policy content of the relevant policy documents in this paper. Using ROSTCM6 software, the content of 16 policies is imported into the text mining database for word segmentation and screening. After effectively removing interference words such as "implement," "promote," and "improve," the high-frequency words are extracted (see Table 2). The document set is segmented, followed by word frequency statistics, which are displayed from high to low frequency, and a social network graph is established (see Fig 5).

**Table 2:** High frequency vocabulary table.

Vocabulary	Frequency	Vocabulary	Frequency	Vocabulary	Frequency
Finance	375	Mechanism	181	Investment	95
Market	351	Data	178	Financing	88
Enterprise	285	Regulation	135	Property Rights	86
Innovation	277	Management	129	Knowledge	78
Technology	257	Trade	114	Industry	76
Development	206	Digital	113	Talent	62
Service	198	Element	100	Quality	52

To explore the operational level of policy tools, based on cybernetics and within the framework of "standard information behavior," this paper studies policy tools [58]. According to the mechanism of action of science and technology financial policies, different keywords are selected from the basic elements of policy orientation to analyze the basic elements of policy tools. Then, through comparison and classification, the main axis and selection codes of policy tools are formed.

Firstly, looking at the distribution of high-frequency words, financial and market vocabulary appears more than 300 times, indicating that the focus of the policy is very clear, reflecting the core idea of the financial marketization trend; the frequency of words such as enterprise, innovation, and technology exceeds 200 times, indicating that the documents focus on execution efficiency, while the remaining frequencies mainly reflect the scope of policy application.

Secondly, based on the Omnia mobilis hypothesis and combining the research findings of Ruiz Estrada et al. [43], related literature, and the results of vocabulary processing from text statistics, it is found that policy texts are centered on "innovation," with finance and technology as the core, deriving keywords such as talent, institutions, markets, and enterprises. Based on the aforementioned research, 9 primary variables and 34 secondary variables are set. The primary variables include: policy timeliness (X1); policy nature (X2); policy content (X3); policy field (X4); target of action (X5); perspective of action (X6); policy system (X7); issuing institution (X8); effectiveness level (X9).

The secondary variables contained within each primary variable are as follows:

1. Policy Timeliness (X1) includes three secondary indicators: (X1:1) Long-term (>5 years); (X1:2) Medium-term (3–5 years); (X1:3) Short-term (1–3 years), which are used to define whether the timeliness of the science and technology financial policy is long-term, short-term, or medium-term.
2. Policy Nature (X2) includes six secondary indicators: (X2:1) Forecast; (X2:2) Suggestion; (X2:3) Identification; (X2:4) Regulation; (X2:5) Description; (X2:6) Guidance. These are used to evaluate whether the policy possesses functions of suggestion, forecast, identification, regulation, description, or guidance.
3. Policy Content (X3) includes six secondary indicators: (X3:1) Finance; (X3:2) Technology; (X3:3) Market; (X3:4) Knowledge; (X3:5) Service Industry; (X3:6) Property Rights. These six indicators are used to evaluate the content included in a science and technology financial policy. Finance refers to financial policies issued by central documents (investment and financing, loans, interest rates, monetary tools, etc.); Technology refers to policies introduced to promote scientific and technological innovation (corporate scientific research, scientific and technological productivity, etc.); Market mainly involves China’s multi-level capital markets; Knowledge refers to systems such as science and technology finance talent and theories; Service Industry refers to the service industries related to finance, such as insurance, accounting, auditing, securities, and other financial services; Property Rights mainly refer to aspects such as the definition of property rights for technology-based enterprises.
4. Policy Field (X4) includes five secondary indicators: (X4:1) Finance; (X4:2) Technology; (X4:3) Talent; (X4:4) Market; (X4:5) Industry, which are used to measure whether a science and technology financial policy involves the fields of finance, technology, talent, market, and industry.
5. Target of Action (X5) includes four secondary indicators: (X5:1) Enterprises; (X5:2) Banks; (X5:3) Government; (X5:4) Other Financial Institutions. These four indicators are used to measure whether a science and technology financial policy involves these entities.
6. Perspective of Action (X6) includes two secondary indicators: (X6:1) Micro; (X6:2) Macro.
7. Policy System (X7) includes four secondary indicators: (X7:1) Clear Objectives; (X7:2) Feasible Scheme; (X7:3) Adequate Basis; (X7:4) Conformity with National Conditions. These four indicators are used to measure whether a science and technology financial policy has clear objectives, an adequate basis, a feasible scheme, and conforms to national conditions.
8. Issuing Institution (X8) includes four secondary indicators: (X8:1) State Council; (X8:2) Ministry of Finance; (X8:3) Central Bank; (X8:4) Securities Regulatory Commission, Banking and Insurance Regulatory Commission, and other financial departments. The data selected in this paper mainly comes from policies enacted at the central level.
9. Effectiveness Level (X9) includes five secondary indicators: (X9:1) Laws and Regulations; (X9:2) Administrative Regulations; (X9:3) Departmental Rules; (X9:4) Normative Documents; (X9:5) Industry Regulations. These five secondary indicators are used to evaluate the effectiveness level of a science and technology financial policy, and the levels of effectiveness decrease in the order listed.

The setup of each level of variables in the index model for the science and technology financial policies is shown in Table 3.

**Table 3:** Variable settings for the PMC-AE index model of science and technology financial policy.

Primary Variable	Secondary Variable
(X1) Policy Timeliness	(X1:1) Long-term (>5 years)
	(X1:2) Medium-term (3–5 years)
	(X1:3) Short-term (1–3 years)
(X2) Policy Nature	(X2:1) Forecast
	(X2:2) Suggestion
	(X2:3) Identification
	(X2:4) Regulation
	(X2:5) Description
	(X2:6) Guidance
(X3) Policy Content	(X3:1) Finance
	(X3:2) Technology
	(X3:3) Market
	(X3:4) Knowledge
	(X3:5) Service Industry
	(X3:6) Property Rights
(X4) Policy Field	(X4:1) Finance
	(X4:2) Technology
	(X4:3) Talent
	(X4:4) Market
	(X4:5) Industry
(X5) Target of Action	(X5:1) Enterprises
	(X5:2) Banks
	(X5:3) Government
	(X5:4) Other Financial Institutions
(X6) Perspective of Action	(X6:1) Micro
	(X6:2) Macro
(X7) Policy System	(X7:1) Clear Objectives
	(X7:2) Feasible Scheme
	(X7:3) Adequate Basis
	(X7:4) Conformity with National Conditions
(X8) Issuing Institution	(X8:1) State Council
	(X8:2) Ministry of Finance
	(X8:3) Central Bank
	(X8:4) Securities Regulatory Commission, Banking and Insurance Regulatory Commission, and other financial departments
(X9) Effectiveness Level	(X9:1) Laws and Regulations
	(X9:2) Administrative Regulations
	(X9:3) Departmental Rules
	(X9:4) Normative Documents
	(X9:5) Industry Regulations

#### Setting of secondary variable parameters

The calculation of the PMC-AE Index Model uses binary for the setting of secondary variable parameters, with efficacy indicators exhibiting progressive and exclusive characteristics. If the content is present in the policy document, it is counted as 1; otherwise, it is counted as 0. The score results of the secondary variable parameters are shown in Table 4.

**Table 4:** Setting of secondary variable parameters.

Primary Variable	Secondary Variable	Parameter Setting
X1	X1:1	Is this science and technology financial policy a long-term policy? Yes = 1, No = 0
	X1:2	Is this science and technology financial policy a medium-term policy? Yes = 1, No = 0
	X1:3	Is this science and technology financial policy a short-term policy? Yes = 1, No = 0
X2	X2:1	Does this science and technology financial policy have forecasting significance? Yes = 1, No = 0
	X2:2	Does this science and technology financial policy have recommendation significance? Yes = 1, No = 0
	X2:3	Does this science and technology financial policy have identification significance? Yes = 1, No = 0
	X2:4	Does this science and technology financial policy have regulation significance? Yes = 1, No = 0
	X2:5	Does this science and technology financial policy have description significance? Yes = 1, No = 0
	X2:6	Does this science and technology financial policy have directional function? Yes = 1, No = 0
X3	X3:1	Does this science and technology financial policy involve financial content? Yes = 1, No = 0
	X3:2	Does this science and technology financial policy involve technological content? Yes = 1, No = 0
	X3:3	Does this science and technology financial policy involve market content? Yes = 1, No = 0
	X3:4	Does this science and technology financial policy involve knowledge content? Yes = 1, No = 0
	X3:5	Does this science and technology financial policy involve service content? Yes = 1, No = 0
	X3:6	Does this science and technology financial policy involve property rights content? Yes = 1, No = 0
X4	X4:1	Does this science and technology financial policy involve finance? Yes = 1, No = 0
	X4:2	Does this science and technology financial policy involve technology? Yes = 1, No = 0
	X4:3	Does this science and technology financial policy involve talent? Yes = 1, No = 0
	X4:4	Does this science and technology financial policy involve market? Yes = 1, No = 0
	X4:5	Does this science and technology financial policy involve industry? Yes = 1, No = 0
X5	X5:1	Is this science and technology financial policy targeted at enterprises? Yes = 1, No = 0
	X5:2	Is this science and technology financial policy targeted at banks? Yes = 1, No = 0
	X5:3	Is this science and technology financial policy targeted at government? Yes = 1, No = 0
	X5:4	Is this science and technology financial policy targeted at other financial institutions? Yes = 1, No = 0
X6	X6:1	Is this science and technology financial policy in the micro domain? Yes = 1, No = 0
	X6:2	Is this science and technology financial policy in the macro domain? Yes = 1, No = 0
X7	X7:1	Does this science and technology financial policy have clear objectives? Yes = 1, No = 0
	X7:2	Are the plans of this science and technology financial policy feasible? Yes = 1, No = 0
	X7:3	Is this science and technology financial policy sufficiently based? Yes = 1, No = 0
	X7:4	Does this science and technology financial policy align with national conditions? Yes = 1, No = 0
X8	X8:1	Is the State Council involved in this science and technology financial policy? Yes = 1, No = 0
	X8:2	Is the Ministry of Finance involved in this science and technology financial policy? Yes = 1, No = 0
	X8:3	Is the Central Bank involved in this science and technology financial policy? Yes = 1, No = 0
	X8:4	Are other financial departments involved in this science and technology financial policy? Yes = 1, No = 0
X9	X9:1	Is this science and technology financial policy a law or regulation? Yes = 1, No = 0
	X9:2	Is this science and technology financial policy an administrative regulation? Yes = 1, No = 0
	X9:3	Is this science and technology financial policy a departmental rule? Yes = 1, No = 0
	X9:4	Is this science and technology financial policy a normative document? Yes = 1, No = 0
	X9:5	Is this science and technology financial policy an industry regulation? Yes = 1, No = 0

#### Establishment of multiple input-output tables

Multiple input-output tables are an optional data analysis framework that can store a large amount of data and measure the variables of science and technology finance innovation policy evaluation from multiple dimensions, reflecting the evolution process of a certain policy. These tables consist of several primary variables and an unlimited number of secondary variables. This article combines the indicators of China’s science and technology finance innovation policy to establish multiple input-output tables (see Table 5).

**Table 5:** Multi-input-output tables.

Primary Variable	X1	X2	X3	X4	X5	X6	X7	X8	X9
**Secondary Variable**	X1:1	X2:1	X3:1	X4:1	X5:1	X6:1	X7:1	X8:1	X9:1
	X1:2	X2:2	X3:2	X4:2	X5:2	X6:2	X7:2	X8:2	X9:2
	X1:3	X2:3	X3:3	X4:3	X5:3		X7:3	X8:3	X9:3
		X2:4	X3:4	X4:4	X5:4		X7:4	X8:4	X9:4
		X2:5	X3:5	X4:5					X9:5
		X2:6	X3:6						

#### Calculation of PMC-AE index

The first step is to calculate the PMC-AE index. The specific steps are: first, place primary and secondary variables into the multiple input-output table; second, calculate the specific values of the secondary variables; third, calculate the specific values of the primary variables; fourth, calculate the PMC-AE index. The calculation steps and methods are shown in Table 6, where in the third and fourth steps of the formula, i stands for a primary variable, i = 1, 2, 3,…, m; j stands for a secondary variable, j = 1, 2, 3,…, n.

**Table 6:** Steps for calculating the PMC-AE index.

Steps	Methods	Formula
Step 1	Establishing links between primary and secondary variables	*Χ* ∼ *Ν*[0, 1]
Step 2	Assign values to secondary variables	*Χ* = {*ΧR*: [0, 1]}
Step 3	Determine the values of primary variables	${Χ}_{i}\left[\sum_{j=1}^{n}\frac{{Χ}_{ij}}{\left({Χ}_{ij}\right)}\right]$
Step 4	Calculate the PMC-AE index	$PMC=\sum_{i=1}^{9}\left({Χ}_{i}\left[\sum_{j=1}^{n}\frac{{Χ}_{ij}}{\left({Χ}_{ij}\right)}\right]\right)$

#### PMC-AE index scoring standards

Based on Table 6, after assigning and calculating values one by one, since this article has selected 9 primary evaluation indicators, according to the evaluation standards of Ruiz Estrada [45], the calculated PMC-AE index ranges between 0–9. The specific values of the PMC-AE index are then classified into levels (see Table 7).

**Table 7:** PMC-AE index score levels.

**Score Range**	0–3.99	4–4.99	5–5.99	6–7.99	8–9
**Rating**	Poor	Acceptable	Good	Excellent	Perfect

#### Drawing of PMC-AE surface

The PMC surface is based on the PMC-AE index, presenting the policy evaluation effect in a graphical form. The purpose of constructing the PMC surface is to reflect the research results more vividly and intuitively. To construct the PMC surface, it is first necessary to build the PMC matrix based on the obtained PMC-AE index (Formula 4). This article sets 9 primary variables, thus forming a third-order matrix. Then, according to the calculation results of Table 6, the PMC surface is drawn, which visually displays the policy evaluation effect.


$$PMC\;(surface)=\left[\begin{array}{ccc} Χ1 & Χ2 & Χ3\\ Χ4 & Χ5 & Χ6\\ Χ7 & Χ8 & Χ9 \end{array}\right]$$ (4)

## Quantitative evaluation of technology finance innovation policies

The fundamental idea behind the PMC index model is not to overlook potential related variables. Subjective selection of policy texts can lead to biases in policy evaluation models. Therefore, when choosing policy samples, it is not necessary to follow a specific pattern, nor is it required to classify policies according to criteria such as issuing institutions or levels of efficiency. The PMC-AE index model is developed through an unsupervised data dimension reduction technique that extracts the data characteristics of the main variables. Regardless of the sample selection, the core variables of the database are preserved. The PMC model can be used to evaluate both national and regional policies. In order to ensure the authoritative and instructive nature of policy text evaluations, this paper, based on the aforementioned considerations, randomly selects five major science and technology finance policies introduced at the central level in recent years for quantitative analysis, which reflects the specific implementation of policy texts. These policies play a major role in better evaluating China’s technology finance innovation policies, and the selected texts are shown in Table 8.

**Table 8:** Five science and technology finance policies issued from 2020 to 2022.

ID	Policies	Year
POL1	Opinions on Building a More Perfect System and Mechanism for Market-Based Allocation of Factors	2020
POL2	Suggestions on Formulating the Fourteenth Five-Year Plan for National Economic and Social Development and the Long-Range Objectives Through the Year 2035	2021
POL3	Action Plan for Constructing a High-Standard Market System	2021
POL4	Financial Technology Development Plan (2022–2025)	2022
POL5	Opinions on Accelerating the Construction of a Unified National Market	2022

Based on the multiple input-output tables and policy texts, variables are set to obtain the multiple input-output tables for the five technology finance innovation policies, as shown in Table 9.

**Table 9:** Multiple input-output tables for the five science and technology finance innovation policies.

Primary Variables	Secondary Variables	POL1	POL2	POL3	POL4	POL5
X1	X1:1	1	1	1	0	1
	X1:2	1	1	1	1	1
	X1:3	0	0	1	1	1
X2	X2:1	1	1	1	1	1
	X2:2	1	1	0	1	1
	X2:3	0	1	1	0	0
	X2:4	1	1	1	1	1
	X2:5	1	1	0	0	1
	X2:6	1	1	1	1	1
X3	X3:1	1	1	1	1	1
	X3:2	1	1	1	1	1
	X3:3	1	1	1	1	1
	X3:4	0	1	0	1	1
	X3:5	1	1	0	1	1
	X3:6	1	1	1	0	1
X4	X4:1	1	1	1	1	1
	X4:2	1	1	1	1	1
	X4:3	1	1	0	1	0
	X4:4	1	1	1	0	1
	X4:5	1	1	1	1	1
X5	X5:1	0	1	1	1	1
	X5:2	0	1	0	0	0
	X5:3	1	1	0	0	1
	X5:4	1	1	1	1	1
X6	X6:1	0	1	1	1	1
	X6:2	1	1	1	1	1
X7	X7:1	1	1	1	1	1
	X7:2	1	1	1	1	1
	X7:3	0	1	0	0	1
	X7:4	1	1	1	1	1
X8	X8:1	1	1	1	0	1
	X8:2	0	0	0	0	0
	X8:3	0	0	0	1	0
	X8:4	0	0	0	0	0
X9	X9:1	0	0	0	0	0
	X9:2	0	0	0	0	0
	X9:3	0	0	0	0	0
	X9:4	1	1	1	1	1
	X9:5	1	1	1	1	1

Scores from the five policy texts were used to construct a neural network model using autoencoder technology, and its parameters were learned. The data fusion process includes two stages. The first stage involves merging the scores of the second-level variables for each strategy to obtain the scores for the nine first-level variables of each strategy. In the second stage, the scores of the nine first-level variables are merged to derive the PMC-AE index for the five policies (see Table 10), which are then ranked according to Table 7.

**Table 10:** PMC-AE index of the policies.

	POL1	POL2	POL3	POL4	POL5	Average
**X1**	0.66	0.67	1.00	0.66	1.00	0.668
**X2**	0.80	1.00	0.66	0.66	0.83	0.794
**X3**	0.83	1.00	0.66	0.83	1.00	0.866
**X4**	1.00	1.00	0.80	0.80	0.80	0.88
**X5**	0.50	1.00	0.50	0.50	0.75	0.65
**X6**	0.50	1.00	1.00	1.00	1.00	0.9
**X7**	0.75	1.00	0.75	0.75	1.00	0.85
**X8**	0.25	0.25	0.25	0.25	0.25	0.25
**X9**	0.40	0.40	0.40	0.40	0.40	0.40
**PMC-AE Index**	5.03	7.32	6.04	5.87	7.03	6.258
**Rating**	Good	Excellent	Excellent	Good	Excellent	/

### Drawing PMC surface charts

**Fig 6:**
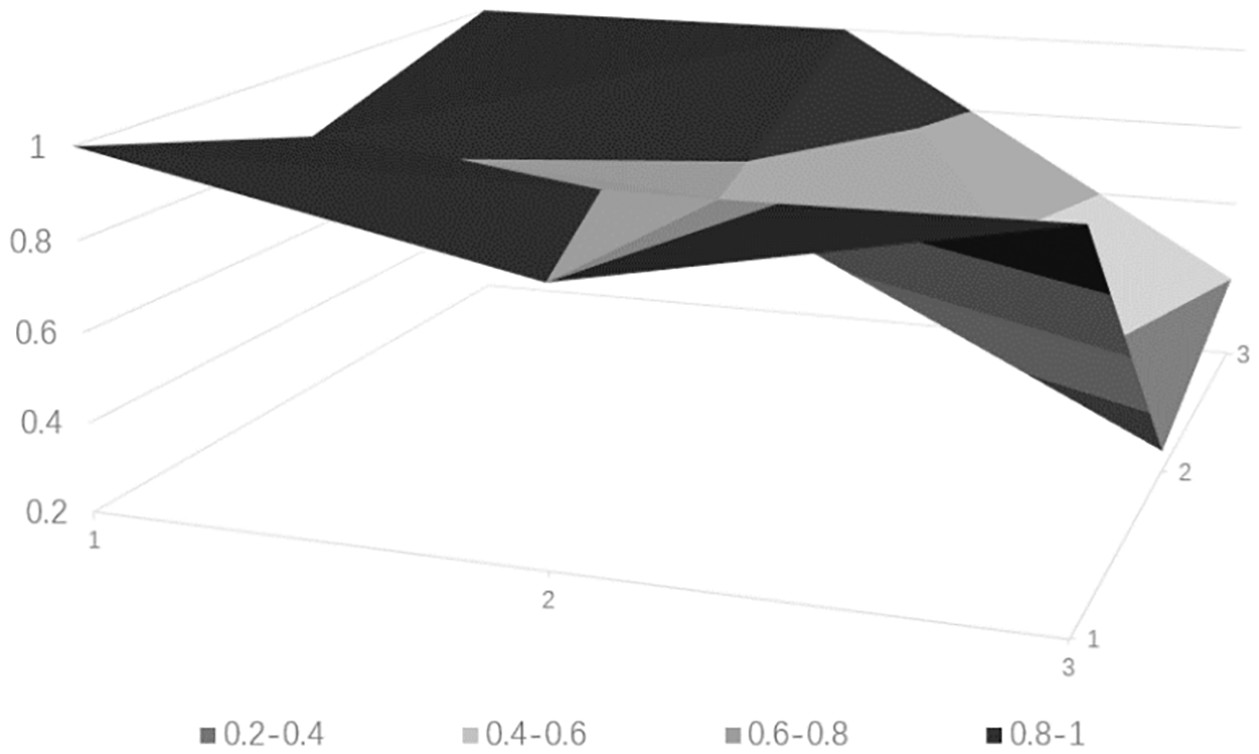


**Fig 7:**
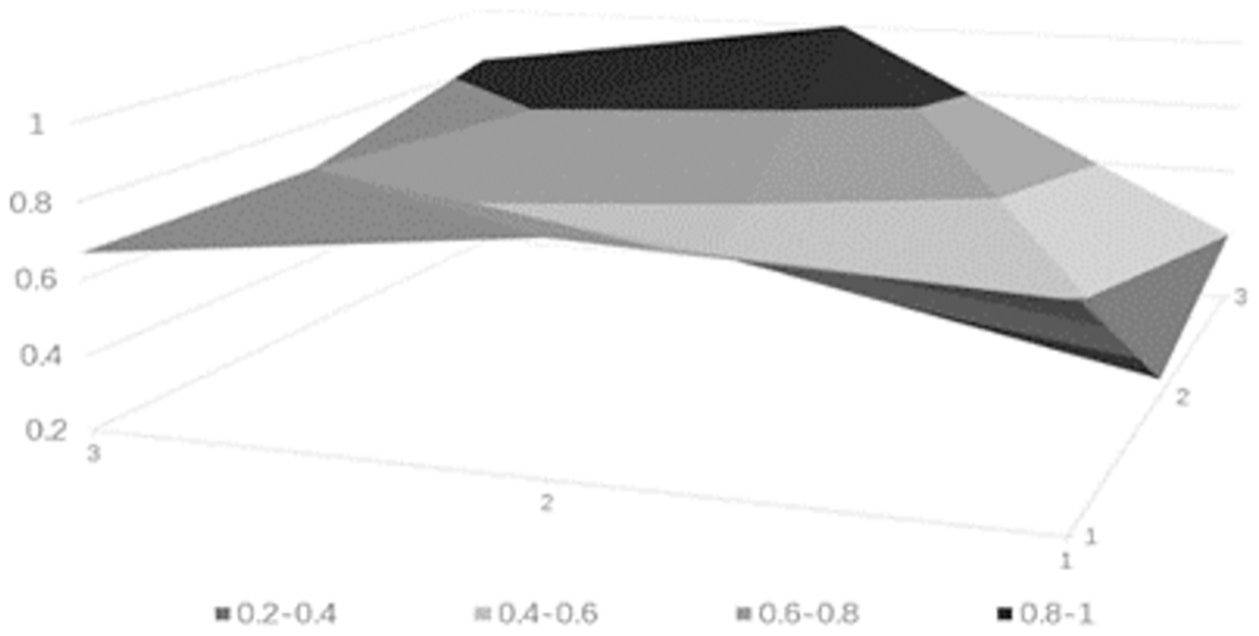


**Fig 8:**
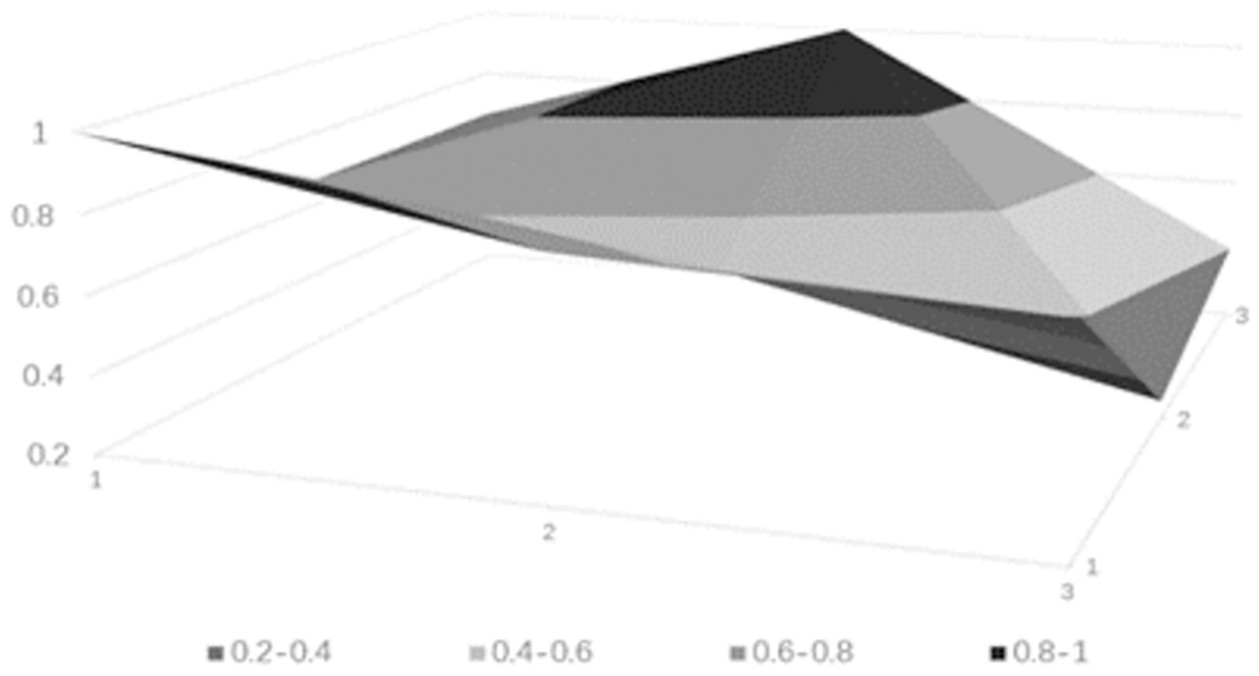


**Fig 9:**
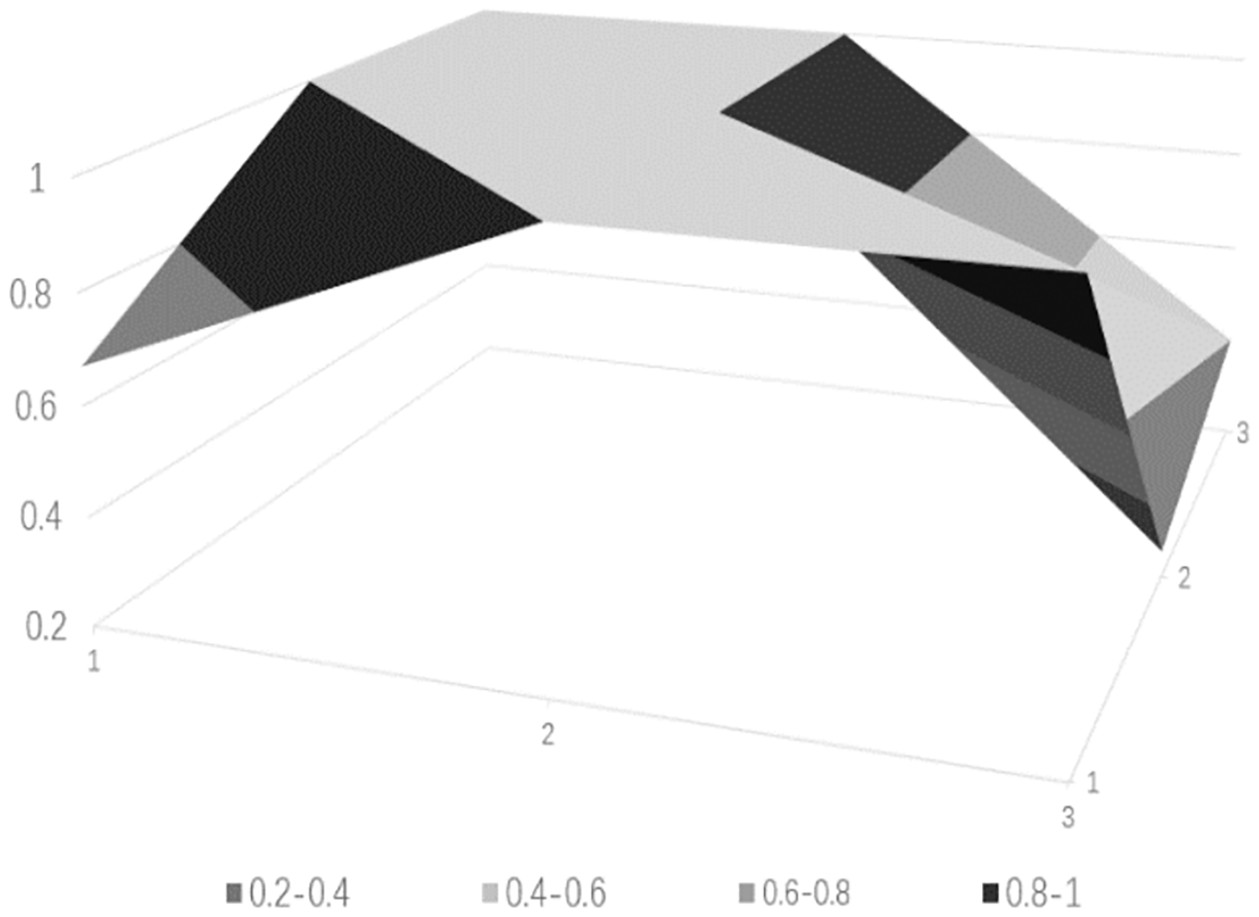


**Fig 10:**
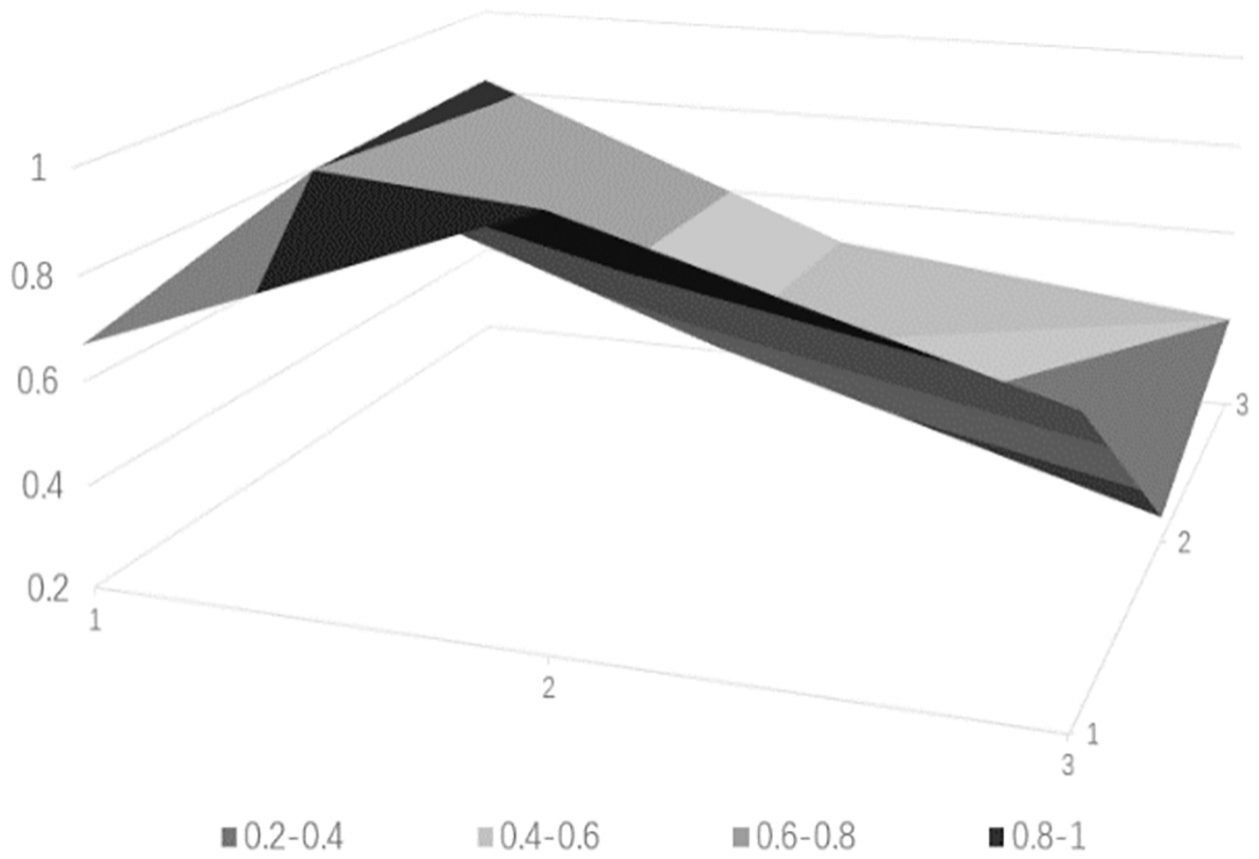


PMC surface charts provide a comprehensive analysis of science and technology finance innovation policies based on intuitive visualization results, with the depth of the concavity negatively correlated with policy ratings. The PMC matrix of the science and technology finance policies can be derived from Table 10 (see Table 11). Based on the aforementioned calculated data, the surface charts for the five science and technology finance policies can be obtained, from Figs 6–10.

**Table 11:** PMC matrix of the five science and technology finance policies.

**Policy**	**POL1**	**POL2**	**POL3**
**PMC Matrix**	$\left[\begin{array}{lll} 0.66 & 0.80 & 0.83\\ 1.00 & 0.50 & 0.50\\ 0.75 & 0.25 & 0.40 \end{array}\right]$	$\left[\begin{array}{lll} 0.66 & 1.00 & 1.00\\ 1.00 & 1.00 & 1.00\\ 1.00 & 0.25 & 0.40 \end{array}\right]$	$\left[\begin{array}{lll} 1.00 & 0.66 & 0.66\\ 0.80 & 0.50 & 1.00\\ 0.75 & 0.25 & 0.40 \end{array}\right]$
**Policy**	**POL4**	**POL5**	
**PMC Matrix**	$\left[\begin{array}{lll} 0.66 & 0.66 & 0.83\\ 0.80 & 0.50 & 1.00\\ 0.75 & 0.25 & 0.40 \end{array}\right]$	$\left[\begin{array}{lll} 1.00 & 0.83 & 1.00\\ 0.80 & 0.75 & 1.00\\ 1.00 & 0.25 & 0.40 \end{array}\right]$	

### Empirical results

The PMC-AE index of the above five policies was calculated, and surface plots were created. Overall, the results indicate that Policy 1 and Policy 4 are at a good level. The remaining three are at an excellent level, and the primary indicators of each of the five sample policies can be intuitively represented through Figs 6 to 10. In terms of the timeliness of Policy X1, it is mainly based on medium- and long-term planning, with a lack of short-term targets. A combination of policies for different time periods should be adopted to meet the needs of various economic development stages. Regarding the nature of Policy X2, the predictive, advisory, regulatory, and guiding functions of the policy are quite excellent, but there is a need for further strengthening in identification and description. Policy X3 is rated as excellent, but there are slight deficiencies in knowledge and property rights, which necessitates focusing on the cultivation of knowledgeable talents in the field of science and technology finance and clearly defining the property rights issues of small and medium-sized science and technology enterprises. In terms of Policy X4’s domain, it basically covers all aspects of China’s economic development. As for the target of Policy X5, there is less policy planning for banking institutions, which should focus on the intermediary role of banks in financial innovation and maximize the support of finance for technology. Policy X6, from the perspective of impact, considers both the micro and macro economy and emphasizes the applicable levels of science and technology finance. Policy X7’s system is based on the current economic development status of China, conforms to the national conditions, with clear and reasonable plans, and effectively promotes innovation in science and technology finance. Regarding the issuing body of Policy X8, there are few issuing bodies, mainly central ones, and there should be a focus on the synergistic effects of policies, combining the actual conditions of central and local governments. For the effectiveness level of Policy X9, it is mainly composed of regulations with lower levels, and there should be an emphasis on improving the effectiveness level of the policies.

From the analysis of individual policies, P2, P3, and P5 are all rated as excellent, while P4 and P1 are rated as good. The average score is in the excellent range, which overall effectively meets the current needs of China’s technological financial development. The PMC index rankings decrease in the following order: P2 > P5 > P3 > P4 > P1. Policy P2 has the highest index with a score of 7.32, indicating the best implementation effect. Only X1 scored below the average, but since the "14th Five-Year Plan" itself is a short to medium-term policy, P2 is considered to be of a particularly excellent level. Policy P5 has an index of 7.03, which is relatively high, but the score for X4 is below average, indicating a need to strengthen the construction of the talent cultivation system. Policy P3 has an index score of 6.04, which is moderate, but scores below the average in aspects X2, X3, X4, X5, and X7, requiring a focus on enhancing the predictive, advisory, and regulatory functions of the policy, and addressing issues in policy content and areas such as knowledge, financial services, and corporate property rights. Policy P4 scored 5.87, which is a good level, but except for X6 and X8, all other items scored below the average. It is suggested to strengthen long-term planning in terms of timeliness, to consider both macro and micro perspectives in terms of the target, and to integrate talent and industrial resources in policy content. Policy P1 scored 5.03, which is relatively low. It performs well in X2, X4, and X8, but scores low in other areas. It is believed that there should be a focus on the coordination between policy and the development of time, and areas such as the target, domain, and content urgently need to be improved.

The detailed analysis of individual policy items shows:

1. Policy 1 is an opinion on market-oriented element allocation, which is primarily reflected in the comprehensive coverage of policy areas (X4:1~X4:5). However, there is a lack of expression regarding the Ministry of Finance, the central bank, and financial institutions (X8:2~X8:4) marked as 0, which may be because the policy-making department does not belong to these financial sectors. The policy is issued by the State Council and is highly directive; legal, administrative regulations, and departmental rules (X9:1~X9:3) are not involved and marked as 0. Other sub-items are at a medium level, with an overall score of 5.03, on the edge of good.
2. Policy 2 is the "14th Five-Year Plan and Long-Term Objectives," mainly reflected in the lack of involvement of financial and fiscal departments (X8:2~X8:4) marked as 0. Almost all other sub-items of the policy are involved, reflecting the comprehensiveness and foresight of the planning objectives, with an evaluation score of 7.32, which is rated as excellent.
3. Policy 3 focuses on the construction of the marketization system, with missing items in the advisory significance (X2:2) and the descriptive meaning of financial technology (X2:5); knowledge content (X3:3); service content (X3:4) not involved and marked as 0; financial policies not targeting banks (X5:2) and enterprises (X5:3) marked as 0; policies still do not involve financial and fiscal departments (X8:2~X8:4) marked as 0. Other sub-items scored high, with an overall policy score of 6.04, on the edge of excellent.
4. Policy 4 is a special plan for financial technology, with missing options in policy identification (X2:3), descriptive meaning (X2:5), property rights content (X3:6), and market requirements (X4:4); no measures for banks (X5:2) and the government (X5:3); lack of clear interpretation in the sufficiency of policy basis (X7:3); the policy formulation does not include the State Council (X8:1), the Ministry of Finance (X8:2), or other financial departments (X8:4) marked as 0. The document is only a normative file with a score of 5.87, in the good range.
5. The national unified large market document does not involve policy identifiability (X2:3), talent elements (X4:3), bank specificity (X5:2), and laws, administrative regulations, and departmental rules (X9:1~X9:3) marked as 0. Other sub-items of the policy are highly directive with high scores. Issued and implemented by the State Council to accelerate the domestic and international dual circulation and build an element circulation system, including guidance on science and technology finance policies, it covers a wide range and has strong directivity with a high evaluation score of 7.03, rated as excellent.

## Discussion

The emergence of technology finance originally stemmed from the national demand for planning and construction of scientific and technological innovation. From the perspective of building an innovative country, the government has, in accordance with the needs of different stages, systematically matched different financial systems with a focus on priority and sequence, committed to achieving the deep integration of financial empowerment of technology and technology finance. A series of policy documents have been formulated to guide the work in various provinces (cities), achieving some results. Through the analysis of 16 documents, we extracted 9 primary variables and 34 secondary variables. Then, we randomly selected 5 national-level texts for evaluation using the PMC-AE model index, which provides a useful reference for assessing the effectiveness of China’s policies on financial innovation in technology. This will promote the improvement of a multi-level technological financial service system and strive to provide strong financial support for the accelerated construction of a modern industrial system.

In the course of our research, we found that China’s policies on financial innovation in technology have played a good guiding role in the specific implementation process. Evaluating five policies, three were rated as excellent and two as good, indicating that the policies were comprehensive and consistent in their effects. In fact, the different combinations of technology finance policies reflect the scientific nature of these policies. The objectives, target subjects, policy content, target goals, and policy system of the policy are clear and have played a significant role in guiding China’s economic development, social progress, and the development of small, medium, and micro enterprises, as well as the financial and technological sectors.

China has set the strategic goal of building an innovative country at the national level, elevating scientific and technological innovation to a national strategic status. It emphasizes the need to guide financial institutions to further optimize products, markets, and service systems according to the different needs of technology enterprises at different development stages, providing a more favorable environment for scientific and technological innovation and a platform for orderly competition in the financial industry. With the successive introduction of science and technology finance policies, the guiding effect of the policies has gradually become apparent. According to data from the People’s Bank of China, by the end of June 2023, the balance of medium and long-term loans in China’s high-tech manufacturing industry reached 2.5 trillion yuan, and the balance of loans for technology-based SMEs was 2.36 trillion yuan. The dividends of policy guidance have gradually emerged, stimulating new momentum for economic growth and achieving a virtuous cycle of finance in the technology industry. Our policy document research shows that X2 (nature of policy), X3 (content of policy), and X6 (target subjects) scored relatively high [53, 57], covering a wide range of content. Overall, China’s policy combination is basically consistent with the model of technological finance innovation and development found in existing research.

Science and technology financial innovation policies are generally aimed at cutting-edge technology innovation projects, which often involve large investments, long cycles, high risk, and significant uncertainty in research and development outcomes. The intrinsic value of technology companies is more reflected in the intangible assets formed by innovation. The development of technology finance urgently needs to strengthen the overall planning and policy coordination at the national level, balance the mechanisms for sharing benefits of innovation across different regions, and require regulatory authorities to view and steadily promote the development of technology finance rationally and objectively, maintaining the sustainable development of the technology finance system. This study indicates that the policy samples scored low in X8 (publishing structure) and X9 (effectiveness level) [56], reflecting a lack of unified scheduling and guidance. Especially, financial institutions in the specific practice process should fully understand the national policy intentions and, under the premise of controllable financial risks, boldly attempt to form a combination of punches with science and technology financial innovation policies.

This study also has some limitations: First, a high PMC-AE index value for a policy model indicates that the policy modeling research has considered a comprehensive set of variables with high consistency, and thus is superior. However, policy modeling is not the same as policy itself, so the appropriateness of transplanting the PMC index model method to policy evaluation is worth discussing. Second, in the target literature, there is ambiguity in the operability of some indicators’ design. For instance, the secondary indicators "finance, technology, market" under the primary indicator "policy content" overlap with the secondary indicators under the primary indicator "policy field" [53]. Despite different focuses in data mining, this can cause interference in the recognition by the autonomous learning module, affecting the accuracy of the evaluation. Third, this paper reviews the policies on technology finance since the 13th Five-Year Plan period, with fewer policies related to the 14th Five-Year Plan period being considered, which may lead to an incomplete analysis of technology finance policies. The use of the PMC model only considers single indicators, lacking a comprehensive grasp and precise quantification of policies. We attempt to address these issues in future similar research by seeking other more advanced methods to avoid deficiencies in research methodology and data processing.

## Research findings and insights

### Research conclusions

The policy of innovation in science and technology finance is an important document guiding the development of China’s economy, and the evaluation of the guiding effectiveness of policy texts is particularly urgent. To achieve this goal, based on 16 representative policy texts on technology finance issued by China’s central authorities from 2015 to 2022, we used text analysis and content analysis methods to extract keyword frequencies, constructed 9 primary variables and 34 secondary variables, and then, for the first time, built the PMC-AE index model for technology finance policy. We randomly selected 5 of the most recently published important policy texts on technology finance for quantitative evaluation. The study shows that from an overall analysis, Policy 1 and Policy 4 are at a good level, while the remaining 3 are at an excellent level. Looking at the policies in detail, there are issues such as unclear legal boundaries of policies, a lack of professional talent in technology finance, weak service consciousness, and the need for stronger financial regulation, with market elements not being clearly defined. Through text analysis, we gained a deeper understanding of the inadequacies in the textual expression of China’s policies on innovation in technology finance. The research results can provide valuable insights for policymakers, help to improve related policies, form sustainable policy guidance texts, and assist in making policy texts more targeted in guiding China’s financial market and rapid economic development. Moreover, as China is in a stage of rapid development, the completeness of policies is conducive to the orderly development of the national economy. This study mainly focuses on the policy texts on innovation in technology finance, revealing the strengths and weaknesses of the policies, which have good guiding effectiveness. The practical results are also consistent with policy evaluation. In the future, national or local governments will also introduce related policies, such as new productive forces, the Internet, AI technology, and other economic entities. Our research can provide references for them and can also be used to evaluate policy texts.

### Policy insights

First, strengthen the construction of the science and technology finance talent system (Policy P5’s score for X4 is below average, which requires strengthening the talent training system). (1) Conduct thorough research on the current implementation of science and technology finance, establish the demand for science and technology finance talent over the next ten years, the employment scenarios for science and technology finance talent, integrate science and technology finance talent into various levels of employers, form a tiered talent scale, guide science and technology finance talent to continuously recharge and learn, master high-tech financial auxiliary facility skills, adapt well to the organic combination of traditional models and Internet+, establish a long-term mechanism, so that financial technology talent can better serve society and improve the efficiency of financial technology policy implementation; (2) Strengthen incentives for science and technology talent, guide employers at the national level to introduce specific measures to incentivize financial technology talent, adopt flexible, attractive, and challenging policy measures to encourage financial technology talent to stay, and equip a series of auxiliary incentive models such as: settlement issues in major cities like Beijing, Shanghai, Guangzhou, and Shenzhen, housing subsidies, salary rewards, etc., insist on implementation, and establish a more comprehensive talent incentive and protection policy.

Second, improve the level of financial services (Policy X2 needs further strengthening in terms of identification and description functions). (1) Policy formulation needs to clearly define the target audience, in response to the current difficulties and high costs of financing for small and medium-sized technology enterprises, introduce a more specific science and technology finance policy system to enhance the feasibility and identifiability of the policy; (2) Fully utilize technological means to aid the efficiency of financial services, increase the empowerment of payment services by technology, explore multi-channel identity verification based on cross-industry data resources, enhance the efficiency of financial service customer identification, and leverage the customer aggregation effect; (3) Enhance the inclusiveness of science and technology finance for the benefit of the people’s livelihood, use different scenarios for publicity, promote the functions and applicable methods of science and technology finance, penetrate customer groups, popularize the convenient functions of science and technology finance, not only play the advantages of the Internet in enterprises and groups but also increase the experience functions in the vast grassroots social groups, empowering every aspect of people’s lives.

Third, improve the coordination between central and local financial policies (There are few issuing institutions in aspect X8). (1) Among the existing policy measures, those with guidance are concentrated at the national level and cover a wide range of content. However, the technology finance field is broad, and there are relatively few instructive and operational documents from various ministries and commissions, leading to a lack of guidance at both macro and micro levels, or in interpretative documents. It is necessary to improve communication between ministries and related functional departments and to formulate practical guiding documents, such as the financial department issuing documents on loans, interest subsidies, tax subsidies, and tax reductions for technology finance enterprises to enhance the practicality of the documents; (2) The current technology finance policies do not provide strong support for local enterprises, small and medium-sized enterprises, and private enterprises. It is necessary to conduct serious research and fully utilize financial derivative tools such as guarantees and trusts to ensure advanced technical means, secure funding, and controlled risks. Financial technology policies should serve the local economy well, strengthening the consistency and coordination between central and local financial policies.

Fourth, strengthen the enforcement of policies protecting intellectual property rights in technology finance (There is a slight lack in the area of knowledge and property rights in policy content X3). (1) Fully utilize market regulation, industrial and commercial, and technical investigative methods for joint enforcement, to punish in a timely manner the false information and methods in technology finance that violate regulations and disrupt the good order of technology finance, maintain the national economic order, define the boundaries of intellectual property rights, build a full-chain intellectual property financial service system, and create an environment that respects intellectual property rights; (2) Increase the punishment for illegal activities in technology finance that have caused significant economic losses, using judicial procedures to compensate first and stop losses in a timely manner.

Fifth, increase the intensity of financial regulation (Policy P3 scores below average in aspects X2, X3, X4, X5, and X7, and needs to focus on strengthening the policy’s predictive, advisory, and regulatory functions). (1) Financial departments should establish market supervision institutions to inspect the implementation of technology finance operations, prepare pre-emptive plans, regularly publish inspection results, grade and rate them, and award credit ratings during the operation of technology finance, advocating for departments and individuals to maintain the order of technology finance operations; (2) Improve the regulatory function system of technology finance, use existing technological means and the authority of big data information to uncover covert illegal activities, enhance full-process regulatory capabilities, and form a joint supervision system with consistent information sharing and coordination between financial departments and law enforcement agencies; (3) Each department should form work reports for routine and emergent events, propose constructive suggestions, and submit them to the financial department to establish basic and general applicable requirements.
